# Parent Questionnaires in the Evaluation of Pre-School Children Referred for Neuropsychiatric Assessment

**DOI:** 10.1007/s10803-021-05080-y

**Published:** 2021-05-18

**Authors:** Mats Cederlund

**Affiliations:** 1 NU Hospital Organisation in Trollhättan, Trollhättan, Sweden; 2 Cereb AB, Södergatan 11D, 25218 Helsingborg, Sweden

**Keywords:** Autism behavior checklist, ESSENCE-Q, Conners abbreviated parent-teacher, Rating scale, Autism spectrum disorders, Pre-school children

## Abstract

One-hundred twenty-four pre-school children referred for assessment to a neuropsychiatric team were included in this study of the Autism Behavior Checklist (ABC), ESSENCE-Q, and Conners Abbreviated Parent-Teacher Rating Scale (CAPRS). All three questionnaires showed a good correlation towards severity of symptoms in ASD. The ABC questionnaire was, as has been shown in earlier research less accurate in identifying individuals with ASD having an IQ within the normal range. However the ESSENCE-Q, and the CAPRS proved to identify children with difficulties needing further assessment regardless of intellectual ability. The CAPRS showed a good correlation to severity in ASD indicating difficulties in the regulation of activity and behavior likely to be connected to ASD in pre-school children.

## Introduction

Autism spectrum disorder (ASD) is a neurodevelopmental disorder that has been estimated to affect 1–2 individuals in 100 according to previous research (e.g., Harrington & Allen, 2014; Zablotsky et al., 2015). ASD has be found to be reliably diagnosed from 2 years of age, however the average age for a diagnosis of ASD ranges from 4 to 6 years worldwide (Mandell et al., 2010). Early detection of ASD has been proposed to improve outcome, and efforts have been undertaken to lower the age at which a diagnosis of ASD is made through screening, and broad-based developmental surveillance as well as ASD directed screening have been used (Broder-Fingert et al., 2018). Previous studies have shown that parents to children with ASD often identify a possible ASD in their child at an age as early as 12–18 months (De Giacomo & Fombonne, 1998; Wimpory et al., 2000). However, their concern is often not taken seriously by Health care professionals (Ryan & Salisbury, 2012). In addition, once referred for neuropsychiatric assessment the children often end up in long waiting lists, which contributes to the delay in diagnosis (Zwaigenbaum, et al., 2016). Zwaigenbaum et al. (2016) showed in their study that children with more advanced language, better adaptive skills and/or milder ASD symptoms were likely to be overlooked by the screening at 18 months, and not diagnosed until approximately 3 years of age.

Over the last decades several screening instruments have been developed with the purpose of identifying children with suspected Autism Spectrum Disorders (ASD), and other neuropsychiatric/neurodevelopmental disorders in need of further assessment at an early age. Screening instruments are developed to facilitate the collection of information needed in the diagnostic process, when assessing a child with a suspected neuropsychiatric disorder, and not as diagnostic tools as such. When interpreting the information received from screening instruments the investigator must be aware of the difficulties the caregivers might experience in relating the specific items included in the questionnaires to their own child’s real-life behaviors. In addition, symptoms related to ASD might be expressed differently depending on the respective child’s specific verbal and non-verbal skills, and temperamental characteristics, which must also be taken into consideration, when interpreting the results from the questionnaires. Level-one screening instruments have been developed for the general (unselected) population, with the aim to identify children at risk of a developmental disorder. Level-two screening instruments have been developed to identify children at risk of ASD, who are already under the observation for developmental concerns, children who failed level-one screening or children that are siblings of children with a known ASD.

The Early Symptomatic Syndromes Eliciting Neurodevelopmental Clinical Examinations-Questionnaire—(ESSENCE-Q), was developed by Professor Christopher Gillberg, at the Gillberg Neuropsychiatric Center (GNC) in Gothenburg, Sweden in 2010 (Gillberg, 2010). This level-one questionnaire was developed to highlight different psychiatric and learning problems experienced by children, particularly in the first 5 years of life, and to ensure that children, who present with early developmental problems are assessed for these impairments (Neville, 2013). The Conners Abbreviated Parent-Teacher Rating Scale (CAPRS), another level-one screening questionnaire, is an abbreviated version of the original Conners questionnaires for teachers and parents, respectively, addressing issues pertaining to difficulties in the regulation of activity, concentration, mood, and behavior, and was originally developed for the evaluation of symptoms related to Attention Deficit Hyperactivity Disorder (ADHD) (Conners, 1969).

The Autism Behavior Checklist (ABC) (Krug et al., 1980), which is a level-two screening questionnaire, focuses on the evaluation of autistic signs and symptoms present in an individual suspected of having an ASD. The ABC is easy to administer, and covers all main problematic areas included in ASD, albeit was originally developed for the assessment of autistic symptoms in individuals with moderate to severe intellectual disability, which might make it less assigned for children with near normal or normal intelligence. From earlier research it has been reported that children with an IQ above 70 tend to score below the cut-off proposed for the ABC, making the authors dispute the diagnostic validity for the ABC in higher intellectually functioning individuals (Volkmar et al., 1988; Wadden et al., 1991). However, in their study Volkmar et al. (1988) found a good correlation across ABC scores and the Vineland Adaptive Behavior Scales, except for the scores related to language (Volkmar et al., 1988).

In this study the author decided to use these three individually quite different questionnaires in order to evaluate different problems present in pre-school children referred for neuropsychiatric assessment. The ESSENCE-Q, which is the most recently developed questionnaire of the questionnaires used, was selected because it has a broader approach addressing neurodevelopmental as well as neuropsychiatric problems, including issues covering, for example, inattention, communication, social interaction, sleep, and feeding problems. The CAPRS was chosen to assess the presence of more “typical” ADHD symptoms such as hyperactivity, difficulties with concentration, impulsivity, mood, and behavior in this group of pre-school children referred for neuropsychiatric assessment, in place of larger and more complex questionnaires primarily developed for the assessment of school children. Finally, the ABC was chosen to evaluate the usefulness of this questionnaire, originally developed to evaluate autistic symptoms and signs in children with moderate to severe intellectual disability, in this group of presumably less intellectually affected pre-school children.

To create a solid base for the gathering of relevant information for the diagnostic process the Diagnostic Interview for Social and Communication Disorders-Eleventh Edition (DISCO-11) was used (Wing, 2006; Wing et al., 2002). In this semi-quantitative instrument information retrieved from the parent(s)/caregiver(s) is combined with the investigating professional’s view of the child. The DISCO-11 was chosen in favor of the Autism Diagnostic Interview-Revised (ADI-R) (Lord et al. (1994) since the DISCO-11, in addition to the large number of items addressing autistic symptoms and signs also has a developmental perspective. Furthermore, these semi-quantitative instruments have shown excellent overall agreement according to the Landis and Koch criteria in earlier research (Landis & Koch, 1977; Nygren et al., 2009).

The primary aim of this study was to assess the ABC, the ESSENCE-Q, and the CAPRS, towards the International Statistical Classification of Diseases and Related Health Problems- 10th revision (ICD-10) ASD diagnoses (World Health Organization (WHO), 1992).

## Materials and Methods

### Participants

All the parent(s)/caregiver(s) of the pre-school children referred consecutively for neuropsychiatric assessment to the Child- and adolescent clinic at the NU Hospital, in Trollhättan, Sweden, from April 2014 to June 2016, were invited by letter to take part in the present study if they knew Swedish well enough to be able to perform the DISCO-11 interview without an interpreter present. Both parents had to sign the consent to take part in the study if custody over the child was shared. All in all, 126 (69%) of the 182 children invited for inclusion in the study agreed to participate. For demographic information concerning the participating children please see Table 1.

**Table 1:** Demographic data concerning all children included in the study (n = 126)

	All children (n = 126)	ICD-10 Autism (n = 89)	ICD-10 Atypical autism/AS (n = 17)	No ICD-10 ASD (n = 18)
Girls (%)	30 (23.8)	19 (15.1)	6 (4.8)	5 (4.0)
Boys (%)*/**	96 (76.2)	70 (55.6)	11 (8.7)	13 (10.3)
Mean age all participants in in months (SD)	54.4 (15.8)	53.8 (16.1)	53.4 (15.8)	57.2 (14.6)
Median (min; max)	55 (11; 94)	56 (11; 94)	48 (30; 83)	56 (24; 85)
Mean age girls in months (SD)	50.9 (15.4)	51.6 (15.8)	48.8 (15.3)	51 (17.2)
Median (min; max)	54.5 (24; 74)	56 (28; 73)	53 (30; 74)	55 (24; 71)
Mean age boys in months (SD)	55.4 (15.8)	54.3 (16.2)	55.8 (15.7)	59.5 (13.4)
Median (min; max)	56 (11;94)	55.5 (11;94)	48 (36;83)	59 (35;85)
Born in Sweden (%)*/**	119 (94.4)	83 (69.7)	16 (13.4)	18 (15.1)
Born abroad (%)	7 (5.6)	6 (4.8)	1 (0.8)	0 (0)
Prematurely born girls (%)	4 (13.3)	3 (10.0)	0 (0)	1 (3.3)
Prematurely born boys (%)*	8 (8.3)	5 (5.2)	2 (2.1)	0 (0)

*One prematurely born boy was not diagnosed due to discontinuation of the study

**One boy born at term was not diagnosed due to discontinuation of the study

### Procedure

The DISCO-11 was completed in 124 individuals (98%). In two children the DISCO-11 interview was not performed because of discontinuation of the study. Despite the original exclusion criteria, one of the DISCO-11-interviews had to be made with an interpreter present because after the child was included in the study the parents were found not to master the Swedish language sufficiently for an independent interview.

At the DISCO-11-interview the ABC, the ESSENCE-Q, and the CAPRS were distributed to the parent(s)/caregiver(s) for completion after the investigator had explained to them how to respond to the questionnaires. At the end of the meeting the questionnaires were returned to the investigator.

At the DISCO-11 interview the Vineland-II Parent/caregiver Rating Form was distributed to the parent(s)/caregiver(s) along with a prepaid envelope to be returned to the investigator. The Vineland II Rating Form, which was chosen instead of the Vineland-II Survey Interview Form due to limited time for the investigator for the performance of the study, was distributed to all but three individuals. Two children were < 24 months of age, and below the age at which the Vineland-II Parent/Caregiver Rating Form, Swedish version, was validated, and the third case concerned the parents mentioned above, who did not to know enough Swedish to complete the Vineland-II Survey Interview Form by themselves. All in all, 117 (97%) of the 121 distributed Vineland-II Survey Interview Forms were returned.

The investigating author, who performed all the 124 DISCO-11-interviews, was trained on the DISCO-11 by Lorna Wing and Judith Gould, and has used the DISCO-11 for several years in research as well as in clinical work. In addition, the investigator has been proven to possess scoring ability on the DISCO-11 equivalent to the investigators responsible for the Swedish version of DISCO-11 at the GNC in Gothenburg Sweden.

### Psychological Evaluation

Psychological assessment was performed in 112 children, and 89 of these individuals were tested with one of the Wechsler scales (i.e. Wechsler Preschool and Primary Scale of Intelligence-Third Edition (WPPSI-III, Wechsler, 2002) (n = 24), Wechsler Preschool and Primary Scale of Intelligence-Fourth Edition (WPPSI-IV, Wechsler, 2012) (n = 55), Wechsler Intelligence Scale for Children–Fourth Edition (WISC-IV, Wechsler, 2003) (n = 5), or Wechsler Nonverbal Scale of Ability (WNV, Wechsler & Naglieri, 2006) (n = 5). For the remaining 6 individuals either the Merrill-Palmer-R, (Roid & Sampers, 2000) (n = 4), or the Bayley Scales of Toddler Development-Third Edition, (Bayley, 2006) (n = 2) was used. The 17 individuals, who were assessed by a psychologist, albeit where no intelligence/developmental test was possible to perform, and the additional twelve individuals, who were not assessed by a psychologist, all had their developmental/intellectual level determined by their presentation at the visit to the clinic, and the information concerning developmental abilities acquired from the DISCO-11 interview.

Concerning intellectual ability, the participating children were organized according to the intellectual ability grading system presented in the DISCO-11: (1) Profound intellectual disability (IQ 0–19), (2) Severe intellectual disability (IQ 20–34), (3) Moderate intellectual disability (IQ 35–49), (4) Mild intellectual disability (IQ 50–69), (5) Intelligence below average (IQ 70–89), (6) Average intelligence (IQ 90–119, (7) Intelligence above average (IQ > 120).

### Expressive Language Level

The respective child’s expressive language ability was scored according to information received from the parent(s)/caregiver(s), and from the child’s presentation at the visit to the clinic using the following scheme: Nonverbal, Single words, Phrases, Undeveloped sentences, and Adequate speech.

### Instruments Included in the Study

#### Diagnostic Interview for Social and Communicative Disorders (DISCO-11)

The DISCO-11 is a semi-structured interview intended for interview with a parent/caregiver, who knows the child well. The DISCO-11 includes a range of items with the intention to detect also milder forms of ASD, and it has been proven to be highly valid for assigning diagnoses in the autism spectrum. In addition, it has a developmental perspective and is designed for use from early childhood (Maljars et al., 2012; Nygren et al., 2009; Wing et al., 2002).

#### Autism Behavior Checklist (ABC)

The ABC was developed by Krug et al. (1980), and describes a series of typical autistic behaviors, and aims to assess the presence of these behaviors in a certain subject. The assessment form consists in 57 items, each of which corresponds to a single score referring to an area of symptoms. Five areas are considered in the original ABC questionnaire: sensory (9 items), relating (12 items), stereotypes and object use (12 items), language (13 items), and self-help and social (11 items). The total score is obtained by adding the scores from the different areas. The items are dichotomous (yes/no), albeit they are assigned weights from 1 to 4p according to their importance for the diagnosis at the original chi-square analyses at the validation of the questionnaire. The maximum possible score is 158 p. According to the original validation a score ≥ 67p is strongly indicative of autism; a score between 53 and 66p indicates a moderate probability of autism; a score between 47 and 52p is considered inconclusive for a diagnosis of autism, and a score below 47p is to be considered as non-autistic. In contrast, Nordin and Gillberg (1996) found a score ≥ 45p to be indicative of autism, and Fernell et al. (2010) reported that even scores below 45p would have to be considered as indicative of autism in very young children without speech.

#### Early Symptomatic Syndromes Eliciting Neurodevelopmental Clinical Examinations-Questionnaire (ESSENCE-Q)

A 12-item level-one questionnaire developed for screening purposes in children in Child Care Centers, as a screening tool of neurodevelopmental disorders or as a parent questionnaire in clinical settings, which contains items concerning the child’s (1) General development, (2) Motor development, (3) Reaction to sensory stimuli, (4) Communicative ability, (5) Activity, (6) Attention, (7) Social interaction, (8) Behavior, (9) Mood, (10) Sleep, (11) Feeding, and, the presence of (12) Absences (“Funny spells”). Each item is scored Yes, Maybe/a little, or No, which translates to 2, 1, and 0p, respectively. The cut-off level for the ESSENCE-Q has been proposed to lay at ≥ 3p, which gained a sensitivity rate of 0.87, and a specificity rate of 0.77 in the validating study by Hatakenaka et al. (2016), and in a more recent clinic-based European multicenter study the authors found a sensitivity rate of 0.96, and specificity rate of 0.84, using the same cut-off score (Stevanovic, et al., 2018).

#### Conners Abbreviated Parent-Teacher Rating Scale (CAPRS)

A 10 item level-one questionnaire based on the 10 most often checked items in the Conners 39 items teacher questionnaire, and the Connners 93 items parent questionnaire (Conners, 1969), in which the child’s behavior concerning the following aspects are scored: (1) Restless or overactive, (2) Excitable, impulsive, (3) Disturbs other children, (4) Fails to finish things started – short attention span, (5) Constantly fidgeting, (6) Inattentive, easily distracted, (7) Demands must be met immediately, easily frustrated, (8) Cries often and easily, (9) Mood changes quickly, and drastically, and (10) Temper outbursts, explosive and unpredictable behavior**.** The items are scored on a four graded Likert-scale: 0p—Not true at all/never, 1p—Just a little, 2p—Quite a bit, 3p—Very much true/very frequently. The original cut-off level presented by Conners was 15p (or a mean item score of ≥ 1.5p), above which a clinically significant hyperactivity was likely to be present (Rowe & Rowe, 1997). In a study of pre-school children by Kadesjö et al. (2001), the CAPRS was found to produce significant difference across a group of children with ADHD, and a control group of typically developing children.

#### Vineland Adaptive Behavior Scales- II Parent/Caregiver Rating Form

A Parent/Caregiver Rating Questionnaire, which offers a comprehensive assessment of adaptive behavior. It covers three major areas (1) Communication (2) Daily Living Skills, an (3) Socialization, and in addition a Motor Skills domain is included. From the scores of these scales a General Adaptive Functioning (GAF) Skills score can be derived (Sparrow et al., 2005).

### Statistical Methods

Descriptive statistics are presented as mean, standard deviation, median, minimum and maximum for continuous variables and as numbers and percentages for categorical variables. For comparison between two groups the Student two-sample T-test was used for continuous variables. Effect sizes were calculated for the total scores of all the used questionnaires across the diagnostic groups. The Spearman correlation coefficient was used for the comparison of the items of the respective questionnaires. All significance tests were two-sided and conducted at the 5% significance level.

## Results

### ABC, ESSENCE-Q, and CAPRS Total Scores

When the participating children were collapsed into groups based on the respective ICD-10 ASD diagnoses/No ICD-10 ASD diagnosis they acquired after the assessment, all three screening instruments used were found to have a significant correlation towards severity of the ASD, with the highest mean scores for the group of children with ICD-10 Autism, and the lowest mean scores for the No ICD-10 ASD diagnosis group. The ABC had the strongest. correlation (r_s_ 0.630), and the CAPRS had the weakest correlation (r_s_ 0.379). However, all the assessed questionnaires showed a significant correlation representing a p level of < 0.001. In addition, correlation across the assessed questionnaires was good: ABC versus ESSENCE-Q (r_s_ 0.698, p < 0.001), ABC versus CAPRS (r_s_ 0.579, p < 0.001), and CAPRS versus ESSENCE-Q (r_s_ 0.625, p < 0.001). The results of the effect sizes for the total scores of the used questionnaires across the ICD-10 ASD diagnostic groups are presented in Table 2.

**Table 2:** Effect sizes for the different questionnaires across the ICD-10 ASD diagnostic groups

Variable/ICD-10 ASD diagnoses	ICD-10 autism n = 89	ICD-10 atypical autism/AS n = 17	No ICD-10 ASD n = 18	ICD-10 autism vs ICD-10 AA/AS effect size	ICD-10 autism vs No ICD-10 ASD effect size	ICD-10 AA/AS vs No ICD-10 ASD effect size
ABC	51.4 (22.6)	26,1 (13.5)	13.4 (8.9)	1.18	1.81	1.11
	51 (7;108)	22 (10;48)	9.5 (2;29)			
ESSENCE-Q	11.4 (4.9)	6.6 (3.0)	4.8 (3.2)	1.02	1.41	0.605
	11 (2;21)	6 (2;12)	4 (2;13)			
Conners	13.8 (8.6)	8.5 (6.4)	6.0 (7.5)	0.647	0.930	0.355
	13 (0;30)	7 (0;23)	2.5 (0;23)			
Vineland II GAF	66.5 (13.4)	73.7 (15.8)	84.5 (13.6)	− 0.517	− 1.34	− 0.739
	65 (45;105)	70.5 (48;106)	86 (61;120)			
	n = 84	n = 16	n = 17			

### ABC Total Score and Subscores

Twenty-five out of the 89 individuals (28.1%) diagnosed with ICD-10 Autism scored ≥ 67p, 18 out of the 89 individuals (20.2%) scored between 53 and 66p, and 8 out of the 89 individuals (9.0%) scored between 47 and 52p. Thus 57.3% of the individuals diagnosed with ICD-10 Autism scored positive or inconclusive on the ABC if the originally presented cut-off scores of the questionnaire were used. If the cut-off of ≥ 45p proposed by Nordin and Gillberg (1996) was used 54 individuals (60.7%) scored above cut-off.

All the ABC subscores, representing the sub-areas sensory reactions, relating, stereotypes and object use, language, and self-help and social, were significantly correlated (p < 0.001) to the severity of the ICD-10 ASD diagnoses (Table 3).

**Table 3:** ABC total score, and subscores, in relation to ICD-10 ASD diagnoses

ABC items/Diagnosis	ICD-10 autism n = 89	ICD-10 atypical autism/AS n = 17	No ICD-10 ASD diagnosis n = 18	Spearman correlation coefficient & p value
Total score mean (SD)	51.4 (22.6)	26.1 (13.5)	13.4 (8.9)	r_s_ 0.630
	51 (7;108)	22 (10;48)	9.5 (2;29)	p < 0.001
Sensory (SD)	6.8 (4.0)	2.9 (3.1)	3.0 (2.8)	r_s_ 0.433
	7 (0;17)	3 (0;10)	3 (0;7)	P < 0.001
Social interaction (SD)	13.0 (7.6)	7.5 (6.5)	2.3 (4.1)	r_s_ 0.499
	14 (0;31)	7 (0;26)	0 (0;15)	P < 0.001
Body (SD)	15.5 (8.6)	5.8 (4.5)	4.4 (3.9)	r_s_ 0.553
	15 (0;34)	7 (0;11)	4 (0;11)	p < 0.001
Language/communication (SD)	6.4 (4.6)	4.6 (3.6)	1.0 (1.6)	r_s_ 0.420
	6 (0;20)	4 (1;13)	2.0 (0;6)	p < 0.001
Social adaptation (SD)	9.6 (5.4)	5.1 (3.3)	2.7 (2.3)	r_s_ 0.492
	10 (0;21)	5 (0;12)	2 (0;7)	p < 0.001

### ESSENCE-Q Total Score and Item Scores

All individuals in this study, except three, who received an ICD-10 Autism diagnosis, scored above the cut-off score for ESSENCE-Q of ≥ 3p as proposed by Hatanaka et al. (2016), indicating a need for further neuropsychiatric/neurodevelopmental assessment.

The vast majority, 9 of the 12 items included in the ESSENCE-Q, had a significant correlation to ICD-10 ASD severity, with a correlation at the p < 0.001 level for six of the twelve items (i.e., *Social interaction, General development, Attention, Sensory reactions, Behavior*, and *Feeding*). However, the items *Communication, Sleep,* and *Absences* failed to produce significant differences across the ICD-10 ASD/No ICD-10 ASD groups (Table 4).

**Table 4:** ESSENCE-Q total score, and subscores, in relation to ICD-10 ASD diagnoses (n = 124)

ESSENCE-Q items/Diagnosis	ICD-10 autism n = 89	ICD-10 atypical autism/AS n = 17	No ICD-10 ASD n = 18	Spearman correlation coefficient & p value
Total score mean (SD)	11.4 (4.9)	6.6 (3.0)	4.8 (3.2)	r_s_ 0.499
Median (min;max)	11 (2;21)	6 (2;12)	4 (2;13)	p < 0.001
General development	1.1 (0.8)	0.5 (0.7)	0.2 (0.5)	r_s_ 0.412
	1.0 (0;2)	0.0 (0;2)	0.0 (0;2)	p < 0.001
Motor development	0.7 (0.8)	0.5 (0.8)	0.2 (0.4)	r_s_ 0.202
	0.0 (0;2)	0.0 (0;2)	0.0 (0;1)	p < 0.025
Sensory reactions	1.0 (0.8)	0.5 (0.6)	0.3 (0.7)	r_s_ 0.312
	1.0 (0;2)	0.0 (0;2)	0.0 (0;2)	p < 0.001
Communication	1.4 (0.9)	1.1 (1.0)	1.4 (0.9)	r_s_ 0.022
	2.0 (0;2)	1.0 (0;2)	2.0 (0;2)	p < 0.805
Activity	1.4(0.8)	0.8 (0.9)	0.8 (1.0)	r_s_ 0.258
	2.0 (0;2)	0.0 (0;2)	0.0 (0;2)	p < 0.004
Attention	1.4 (0.8)	1.1 (0.7)	0.6 (0.8)	r_s_ 0.328
	2.0 (0;2)	1.0 (0;2)	0.0 (0;2)	p < 0.001
Social interaction	1.4 (0.8)	0.9 (0.8)	0.4 (0.6)	r_s_ 0.442
	2.0 (0;2)	1.0 (0;2)	0.0 (0;2)	p < 0.001
Behavior	0.9 (0.9)	0.4 (0.7)	0.1 (0.5)	r_s_ 0.349
	1.0 (0;2)	0.0 (0;2)	0.0 (0;2)	p < 0.001
Mood	0.3 (0.6)	0.0 (0.0)	0.0 (0.0)	r_s_ 0.233
	0.0 (0;2)	0.0 (0.0)	0.0 (0.0)	p = 0.009
Sleep	0.8 (0.9)	0.5 (0.8)	0.4 (0.9)	r_s_ 0.156
	0.0 (0;2)	0.0 (0;2)	0.0 (0;2)	p = 0.085
Feeding	1.0 (0.9)	0.5 (0.7)	0.2 (0.4)	r_s_ 0.375
	1.0 (0;2)	0.0 (0;2)	0.0 (0;1)	p < 0.001
Absences	0.2 (0.5)	0.0 (0.0)	0.1 (0.5)	r_s_ 0.084
	0.0 (0;2)	0.0 (0.0)	0.0 (0.0)	p = 0.356

### CAPRS Total Score and Item Scores

The ICD-10 Autism group had a mean score on CAPRS that came very close to the 15p cut-off level for the questionnaire originally proposed by Conners where a clinically significant hyperactivity was likely to be present. All in all, 44 of the 89 individuals (49.4%) with a diagnosis of ICD-10 Autism scored ≥ 15p on the CAPRS. In contrast, nineteen individuals (21.3%) scored 5p or less on the CAPRS questionnaire.

All the items included in the CAPRS items were correlated to the severity of ICD-10 ASD diagnoses. The items *Easily frustrated*, and *Impulsive,* were the items with the strongest correlation, and *Restless*, and *Cries often* were the items with the weakest correlation towards the ICD-10 ASD diagnoses (Table 5).

**Table 5:** CAPRS total score, and subscores, in relation to ICD- 10 ASD diagnoses (n = 124)

CAPRS/Diagnosis	ICD-10 autism n = 89	ICD-10 atypical autism/AS n = 17	No ICD-10 ASD n = 18	Spearman correlation coefficient & p value
Total score mean (SD)	13.8 (8.6)	8.5 (6.4)	6.0 (7.5)	r_s_ 0.358
Median (min;max)	13 (0;30)	7 (0;23)	2.5 (0;23)	p < 0.001
Restless	1.8 (1.2)	1.2 (1.2)	1.2 (1.3)	r_s_ 0.207
	2.0 (0;3)	1.0 (0;3)	0.5 (0;3)	p = 0.021
Impulsive	1.6 (1.2)	0.8 (1.0)	0.6 (1.0)	r_s_ 0.325
	2.0 (0;3)	1.0 (0;3)	0.0 (0;3)	p < 0.001
Disturbs other	1.1 (1.2)	0.6 (0.7)	0.4 (0.8)	r_s_ 0.255
	1.0 (0;3)	0.0 (0;2)	0.0 (0;3)	p = 0.004
Fails to finish tasks	1.1 (1.2)	1.2 (1.2)	0.7 (1.0)	r_s_ 0.302
	2.0 (0;3)	1.0 (0;3)	0.0 (0;3)	p = 0.001
Fidgeting	1.7 (1.2)	0.6 (1.0)	0.5 (0.8)	r_s_ 0.241
	1.0 (0;3)	0.0 (0;3)	0.0 (0;2)	p = 0.007
Inattentive	1.3 (1.2)	1.1 (1.1)	0.7 (1.0)	r_s_ 0.248
	1.0 (0;3)	1.0 (0;3)	0.0 (0;3)	p = 0.005
Easily frustrated	1.4 (1.2)	0.9 (1.0)	0.8 (1.2)	r_s_ 0.342
	2.0 (0;3)	1.0 (0;3)	0.0 (0;3)	p < 0.001
Cries often	1.8 (1.1)	0.6 (0.9)	0.4 (0.9)	r_s_ 0.213
	1.0 (0;3)	0.0 (0;3)	0.0 (0;3)	p = 0.017
Mood changes	1.1 (1.2)	1.1 (1.3)	0.4 (0.9)	r_s_ 0.255
	1.0 (0;3)	0.0 (0;3)	0.0 (0;3)	p = 0.004
Temper outbursts	1.1 (1.3)	0.4 (0.8)	0.3 (0.8)	r_s_ 0.271
	0.0 (0;3)	0.0 (0;3)	0.0 (0;3)	p = 0.002

### Intellectual Ability

The mean ABC score was significantly correlated to intellectual ability with scores decreasing from the intellectual ability group 3 (IQ 35–49) to the intellectual level group 7 (IQ > 120). However, the ESSENCE-Q, and the CAPRS did not show the same correlation to intellectual ability as did the ABC (Table 6).

**Table 6:** ABC, ESSENCE-Q, and CAPRS mean scores, and Vineland-II GAF mean standard score in relation to intellectual ability for all diagnosed participants in the study (n = 124)

Questionnaires/Intellectual level*	Intelligence group 3 n = 4	Intelligence group 4 n = 29	Intelligence group 5 n = 42	Intelligence group 6 n = 44	Intelligence group 7 n = 5	Spearman correlation coefficient & p value
ABC	61.0 (23.6)	51.0 (23.9)	42.1 (27.0)	38.6 (21.6)	13.8 (6.7)	r_s_ 0.284
	59.5 (35;90)	51 (10;98)	36.5 (2;108)	36.5 (7;87)	12 (6;24)	p = 0.001
ESSENCE-Q	13.5 (5.7)	10.1 (5.4)	10.4 (5.5)	9.4 (4.6)	4.2 (2.3)	r_s_ 0.167
	16 (5;17)	11(2;19)	10 (2;21)	9 (2;18)	4 (2;8)	p = 0.063
CAPRS	12.5 (10.7)	10.3 (8.9)	12.3 (9.1)	13.9 (8.0)	5.2 (5.8)	r_s_ 0.087
	10.5 (3;26)	7 (0,28)	12 (0;27)	12.5 (0;30)	2 (0;14)	p = 0.337
Vineland-II GAF mean standard score	60.8 (15.6)	60.6 (10.4)	67.4 (13.5)	75.7 (12.3)	100.2 (15.2)	r_s_ 0.513
	54 (51;84)	59 (45;90)	66 (47;92)	75 (49;105)	105 (83;120)	p < 0.001

*No children qualified for intelligence groups 1 or 2

### Expressive Language Level

In the ICD-10 Autism group 24.6% of the individuals were nonverbal or only used single words for communication, 10% used phrases, 32.6% was found to use undeveloped sentences, and 32.6% had adequate speech. For the children belonging to the ICD-10 Atypical autism/AS group the respective percentages were 17.6%, 11.8%, 17.6%, and 53.0%, and for the No ICD-10 ASD diagnosis group, the same percentages were 16.7%, 0%, 38.9%, and 44.4%, respectively.

### Adaptive Behavior

All the assessed questionnaires were significantly correlated to Vineland-II GAF mean standard score: CAPRS r_s_ 0.339, p < 0.001; ESSENCE-Q r_s_ 0.500, p < 0.001; and ABC r_s_ 0.587, p < 0.001. A higher mean score on the questionnaires was correlated to a lower adaptive ability score.

## Discussion

In this study all the used questionnaires (the ABC, the ESSENCE-Q, and the CAPRS), were found to correlate to the severity of ICD-10 ASD diagnoses, with the highest scores for the group of children with ICD-10 Autism, lower for the group of children with ICD-10 Atypical autism/AS, and lowest for the group of children, who did not receive an ICD-10 ASD diagnosis at the assessment. In addition, all the used questionnaires showed a good correlation across each other.

The ABC mean score correlated to intellectual ability, with the highest mean score for the group of individuals with the lowest intellectual ability as would be expected from earlier research where individuals with an IQ > 70 were more likely to score below the cut-off for the questionnaire, and hence being falsely judged not having an ASD (e.g., Volkmar et al., 1988; Wadden et al., 1991). The ABC mean score for the participating children was lower than the originally presented cut-off score of 67 points where a diagnosis of Autism is strongly indicated, albeit above the cut-off-scores of ≥ 45p presented by Nordin and Gillberg (1996). However, a large proportion (almost 40%) of the individuals diagnosed with an ICD-10 ASD diagnosis in the study had an ABC score even below a cut-off score ≥ 45p, which could at least partly be explained by the relatively good intellectual ability present in the assessed group, where the mean IQ for the group was in the so called “Below average” IQ range (i.e., IQ 70–89). In addition, 21.3% of the children with an ICD-10 Autism diagnosis were non-speaking or only used single words for communication with the consequence that the items concerning communication were more likely not to be scored for these children, hence leading to lower total scores on the ABC for these children. This was in accordance with the findings in the study by Fumagalli Marteleto and Marcondes Pedromonico (2005), and Fernell et al. (2010).

All ABC subscores were correlated to severity in ICD-10 ASD diagnosis. The subscores for *Body* and *Social adaptation* displayed the most pronounced correlation, and the subscore for *Language* showed the weakest correlation, however all subscores presented with a p level of < 0.001.

In contrast to the ABC, the ESSENCE-Q, and the CAPRS were not significantly correlated to intellectual ability, indicating these questionnaires to be more useful in the evaluation of neuropsychiatric symptoms in children regardless of intellectual ability compared to the ABC.

The results on the ESSENCE-Q were in line with earlier research validating the questionnaire (Gillberg, 2010; Hatakenaka et al., 2016; Stevanovic et al., 2018), where a score of ≥ 3p was considered to indicate a need for further assessment, and only three children (3.4%) included in this study, who acquired an ICD-10 ASD diagnosis, scored below this cut-off level.

The highest item scores for the ESSENCE-Q were found for *Social interaction* followed by *General development*, as would be expected from the clinical perspective. The items *Communication, Sleep*, and *Absences* showed no significant difference across the assessed groups. In the case of *Communication* this might reflect the fact that speech and language problems, which often is a marker for the need for further neuropsychiatric assessment, was the reason for the referring of the child for neuropsychiatric assessment. However, since the children with communication problems, who did not fulfill enough criteria for an ICD-10 ASD diagnosis, ended up in the No ICD-10 ASD diagnosis group, and from there hence conspiring to the absence of a significant correlation across the diagnostic groups concerning the *Communication* item at the assessment.

Interestingly the CAPRS, which was originally developed as a screening instrument for ADHD, was also correlated to severity in ASD, with higher mean scores for the group of children with ICD-10 Autism compared to both the other assessed groups (ICD-10 Atypical autism/AS, and No ICD-10 ASD diagnosis), which speaks in favor of considering early signs such as hyperactivity, and difficulties in finishing tasks, to be more related to autism than to ADHD in pre-school children. The item scores for the CAPRS were all significantly correlated across the ICD-10 ASD diagnoses/No ICD-10 ASD diagnosis groups, albeit from this relatively small material it is not adequate to discuss, which of the items being more ASD or ADHD loaded.

## Conclusions

The result from this study speaks in favor of using all the three assessed questionnaires, the ABC, the ESSENCE-Q, and the CAPRS, in the assessment of pre-school children going through a neurodevelopmental/neuropsychiatric assessment. However, the result from the study confirms previous research data indicating that children with an IQ above 70 often score below the originally validated cut-off levels for the ABC questionnaire. This must be taken into consideration when deciding to use the ABC questionnaire, as well as how to interpret the results of the ABC scoring. The ESSENCE-Q did not show the same correlation to IQ as did the ABC, which is preferable in the screening of children with neurodevelopmental/neuropsychiatric problems, and when the proposed cut-off for the questionnaire was used it identified all but three of the children receiving an ICD-10 Autism diagnosis in the study. In addition, the CAPRS, which originally was developed for the assessment of symptoms relating to ADHD, correlated to severity in ICD-10 ASD diagnoses, indicating that symptoms such as hyperactivity, impulsivity and temper tantrums tend to be related to severity of ASD in pre-school children.

### Limitations

Since this is a comparably small study further studies most be performed using the questionnaires to validate if the results from this study are applicable to larger groups of pre-school children with ASD.
